# It’s Better to Start with a Hug: Technique for Reverse Shoulder Arthroplasty for Three- and Four-Part Proximal Humeral Fractures

**DOI:** 10.3390/jcm15218348

**Published:** 2026-10-27

**Authors:** Francesco Franceschi, Matteo La Verde, Luca Saccone, Antonio Caldaria, Alice Patrignani, Gian Mauro De Angelis D’Ossat, Luca La Verde, Alessio Palumbo, Angelo Baldari

**Affiliations:** 1 Department of Orthopaedic and Trauma Surgery, San Pietro Fatebenefratelli Hospital, 00189 Rome, Italydoc.angelobaldari@gmail.com (A.B.); 2 Faculty of Medicine and Surgery, Link Campus University, 00165 Rome, Italy; 3 II Orthopaedic and Traumatology Clinic, IRCCS Istituto Ortopedico Rizzoli-DIBINEM, University of Bologna, 40100 Bologna, Italy; 4 Faculty of Medicind and Surgery, IUniCamillus-Saint Camillus International University of Health Sciences, 00131 Rome, Italy

**Keywords:** reverse shoulder arthroplasty, proximal humeral fracture, tuberosity fixation, cementless stem, surgical technique

## Abstract

**Background:** Reverse shoulder arthroplasty (RSA) is increasingly employed for managing complex three- and four-part proximal humeral fractures (PHFs) in elderly patients. Tuberosity healing and implant stability remain critical challenges, particularly in uncemented RSA. We describe a novel surgical technique—the “Hug Technique” for uncemented RSA in acute three- and four-part PHFs, designed to preserve metaphyseal bone stock, avoid tuberosity osteotomy, and optimize tuberosity healing. Additionally, we evaluated the functional outcomes of our novel surgical technique. **Methods:** A single-center retrospective cohort study was conducted on patients treated with the “Hug Technique” between January 2023 and January 2025. Inclusion criteria were acute displaced three- or four-part PHFs, age > 65 years, intact deltoid function, and ≥12 months of follow-up. All procedures were performed by a single surgeon using a standardized reverse shoulder implant. Clinical outcomes, functional scores, radiographic findings, complications, and implant survivorship were assessed. **Results:** A total of 14 patients were included, with a mean age of 74.8 years and a mean follow-up of 14.2 months. At final follow-up, the mean postoperative Constant score was 68.4, and the mean ASES score was 81.6. Mean active forward flexion reached 132°, external rotation 28°, and internal rotation reached the L3 vertebral level in most patients. Anatomical or consolidated tuberosity healing was observed in 78.5% of cases. No cases of stem loosening were observed. Complications occurred in 2 patients, including one case of deep periprosthetic infection and one case of anterior dislocation. **Conclusions:** The “Hug Technique” represents a feasible surgical strategy for cementless RSA in complex PHFs. Preliminary clinical and radiographic results appear promising. Further prospective comparative studies are required.

## 1. Introduction

In recent years, the indication for reverse shoulder arthroplasty (RSA) has extended beyond rotator cuff arthropathy to encompass the management of complex acute proximal humeral fractures (PHFs). Several studies have evaluated the long-term clinical and functional outcomes of RSA performed for PHF in comparison to elective procedures for degenerative conditions, showing consistent long-term implant survival and functional results [1,2,3]. These encouraging results have contributed to a substantial increase in the use of RSA for fracture management [4].

It is now well established that in patients over the age of 70 with three- or four-part PHF, primary RSA leads to superior patient-reported outcomes and greater range of motion compared to open reduction and internal fixation (ORIF) using an angular stable locking plate [4,5,6]. A meta-analysis of seven studies found that elderly patients with healed tuberosities after RSA exhibited superior active forward flexion, external rotation, and external rotation strength [7].

In an effort to manage bone loss and increase implant stability, RSA for PHF has often been performed with a cemented stem fixation, as the fractured proximal humerus was not considered to provide adequate support for metaphyseal fixation [8,9]. Although initially cementing the humeral stem allows precise restoration of height and anatomical version, providing solid primary stability, it has been shown to be associated with longer operating times, increased thromboembolic risk, higher costs, possible interference with tuberosity healing due to exothermic reaction, and increased complexity of revision surgery, which may require a humeral osteotomy and carries risks of periprosthetic fracture and bone loss [10,11].

For these reasons, cementless RSA has garnered growing interest, with numerous studies reporting favourable and reproducible clinical and radiographic results similar to those observed with traditional cemented approaches [12,13,14]. Innovations in implant design, in particular the development of shorter stems with porous-coated surfaces for metaphyseal fixation, have introduced several advantages including greater preservation of bone reserve, easier implant revision or removal, and better load distribution on the proximal humerus [15,16]. These properties may help reduce stress-shielding phenomena such as radiolucent line formation and premature component loosening [11].

Achieving reliable metaphyseal fixation with an uncemented stem in three- and four-part proximal humeral fractures remains surgically challenging, largely due to the complexity of intraoperative tuberosity management. Notably, anatomical healing of the tuberosities has been identified as a crucial determinant of functional recovery following reverse shoulder arthroplasty for acute fractures, with a significant impact on postoperative external rotation and shoulder strength [7].

The aim of this study is to describe the “Hug Technique”, a novel surgical technique for cementless RSA in acute three- and four-part proximal humeral fractures designed to avoid tuberosity osteotomy and preserve metaphyseal bone stock. In addition, we evaluated the preliminary clinical and radiographic outcomes of this technique in a consecutive case series of elderly patients treated for acute PHFs.

## 2. Materials and Methods

### 2.1. Study Design

This single-center retrospective case series was conducted at San Pietro Fatebenefratelli Hospital in Rome (Italy). The study protocol was approved by the Ethics Committee of UniCamillus–Saint Camillus International University of Health Sciences, Rome, Italy (0598/2024), and the study was conducted in accordance with the Declaration of Helsinki. Written informed consent was obtained from all participants prior to surgery. All surgical procedures were performed by the same senior orthopedic surgeon (F.F.), specialized in shoulder arthroplasty.

**Figure 1:**
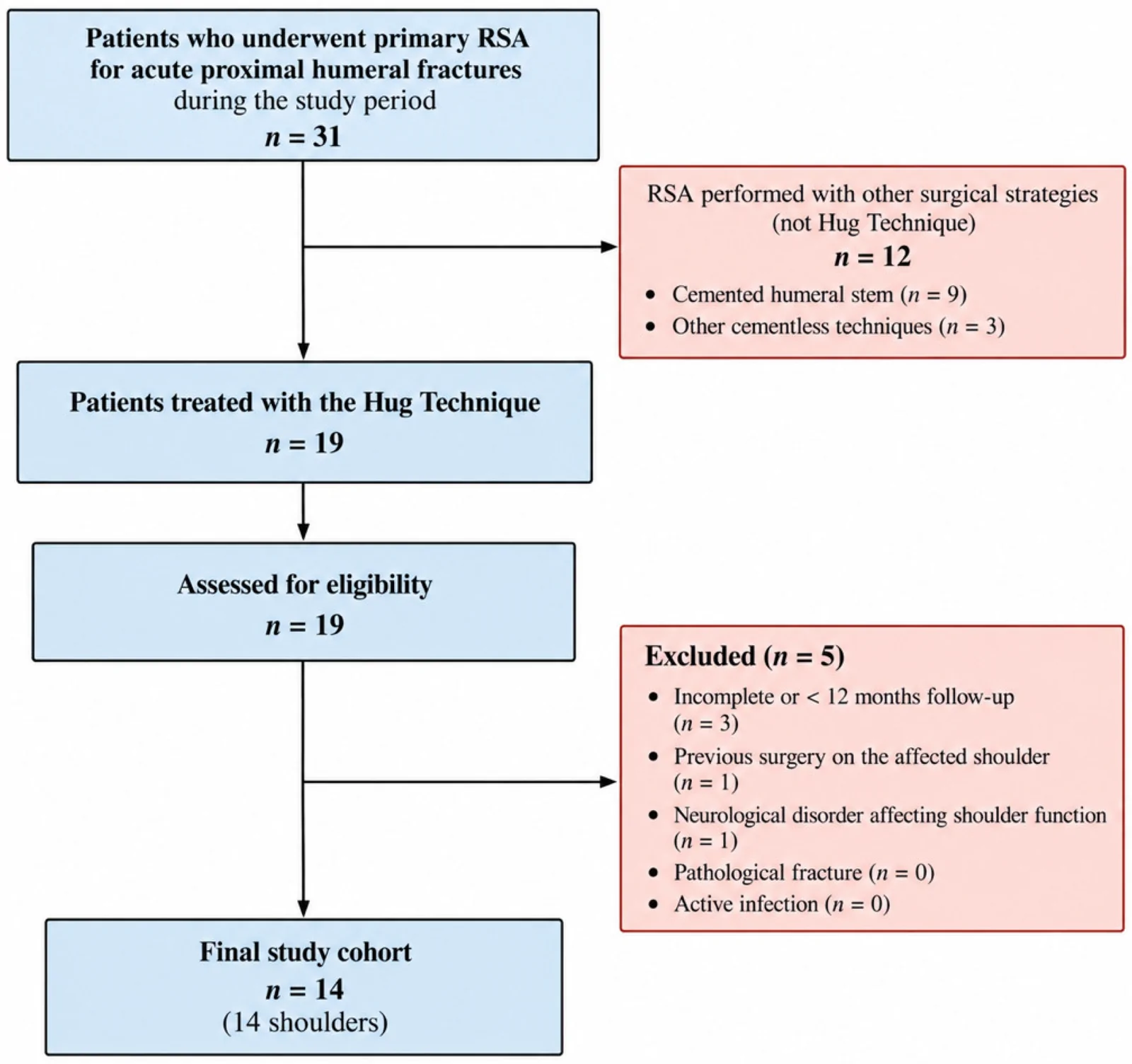
Flowchart of the patient selection process.

All patients who underwent primary reverse shoulder arthroplasty (RSA) for an acute proximal humeral fracture at the study institution between June 2024 and January 2025 were reviewed. During the study period, 31 patients underwent RSA for an acute proximal humeral fracture. The “Hug Technique” was used in 19 of these patients, whereas the remaining 12 patients were managed using alternative surgical strategies and were therefore not considered for inclusion in the present series. Patients treated with the “Hug Technique” were considered eligible for the study if they were older than 65 years, presented with an acute displaced three- or four-part proximal humeral fracture according to the Neer classification, underwent cementless RSA within 3 weeks of injury, and had a minimum clinical and radiographic follow-up of 12 months. Patients presenting with fracture sequelae or pathological fractures, as well as those with previous surgery on the affected shoulder, neurological disorders potentially affecting shoulder function, active infection, or incomplete clinical or radiographic follow-up, were excluded. Of the 19 patients treated with the “Hug Technique”, 5 did not meet the eligibility criteria and were excluded from the final analysis. Three patients had an incomplete or less than 12-month clinical and radiographic follow-up, one had undergone previous surgery on the affected shoulder, and one had an associated neurological disorder potentially affecting shoulder function. Accordingly, the final study cohort consisted of 14 patients (14 shoulders). The patient selection process is summarized in Figure 1.

Clinical evaluation included Constant–Murley score, American Shoulder and Elbow Surgeons (ASES) score, Subjective Shoulder Value (SSV), active range of motion, and complications.

### 2.2. Radiographic Assessment

Tuberosity healing was assessed using standard plain radiographs, including anteroposterior, scapular Y, and axillary views. All radiographs were independently evaluated by two orthopedic surgeons, and no disagreement was observed between the two assessors regarding the classification of tuberosity healing. The final assessment was performed at a minimum follow-up of 12 months. Tuberosity healing was classified as anatomical healing, malunion, or resorption/nonunion. Anatomical healing was defined as radiographic consolidation of the tuberosities in an anatomical or near-anatomical position relative to the humeral component, with restoration of continuity between the tuberosities and the proximal humerus. Malunion was defined as radiographic consolidation of the tuberosities in a displaced or non-anatomical position. Resorption/nonunion was defined as the absence of radiographic consolidation, persistent separation of the tuberosity from the proximal humerus, or partial or complete radiographic resorption of the tuberosity at final follow-up.

### 2.3. Indications

Treatment selection for three- and four-part PHFs depends on fracture pattern, bone quality, rotator cuff status, patient functional demand, and the possibility of obtaining stable fixation [17]. Current evidence suggests that, in elderly patients with displaced complex PHFs, RSA provides superior functional outcomes and lower reoperation rates compared with ORIF [18]. It has been proven that two-part fractures and proximal humeral fracture sequelae (PHFs) can be effectively managed with RSA using techniques that preserve the metaphyseal bone stock and avoid tuberosity osteotomy [19,20,21]. The technique described in this study, which avoids tuberosity osteotomy and preserves the metaphyseal bone, may expand the indications for RSA to include more complex three- and four-part fractures.

### 2.4. Surgical Technique

**Figure 2:**
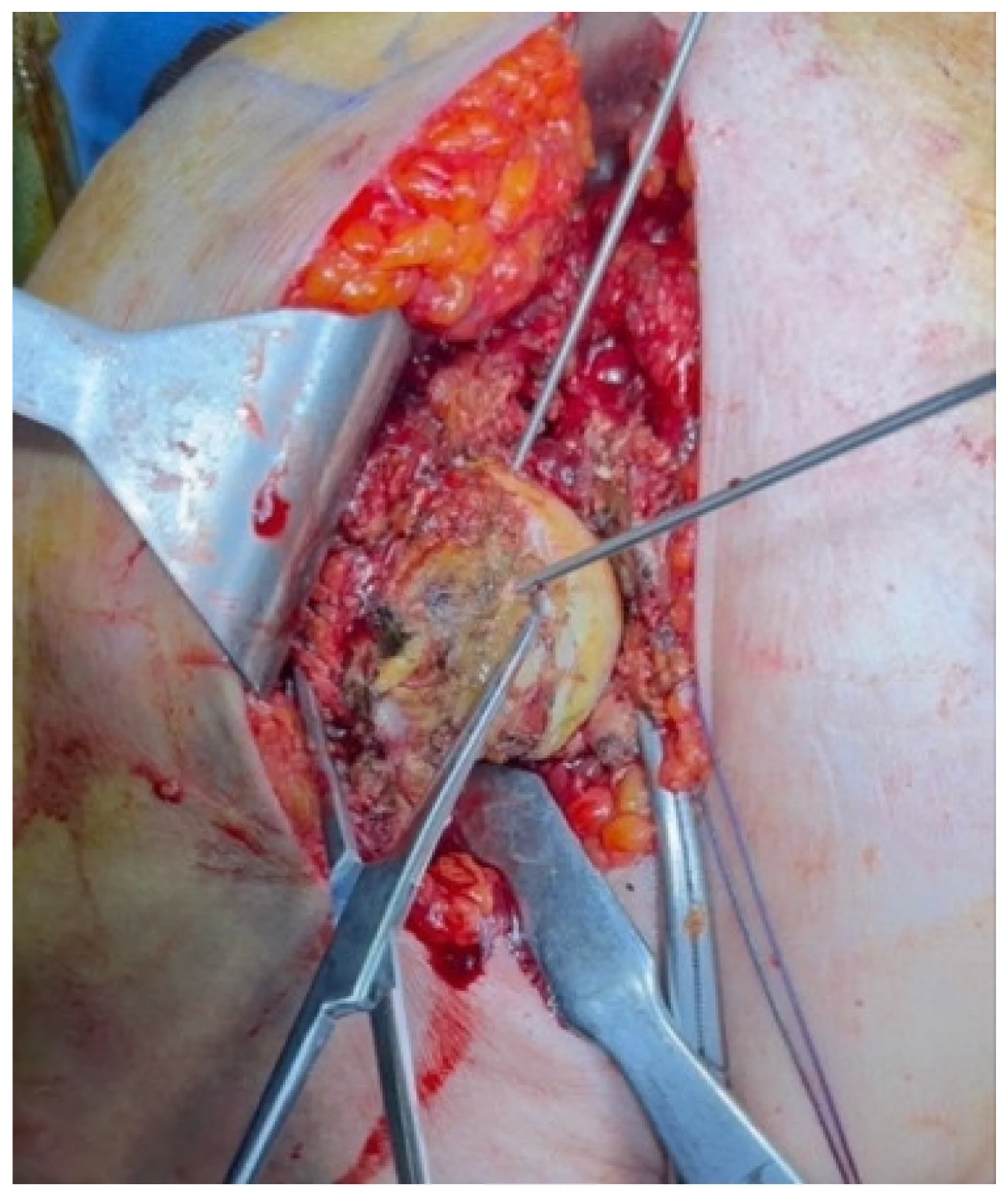
Following reduction of the fragments with a Bernhard towel clamp, the tuberosities are fixed in place using two or more metallic Kirschner wires.

Initial clinical evaluation should confirm the integrity of the axillary nerve, assess deltoid muscle function, and ensure the skin envelope is intact. All procedures were performed with the patient in the beach-chair position, with the operative arm secured in a limb positioner (TRIMANO, Arthrex Inc., Naples, FL, USA), under general anesthesia combined with an interscalene nerve block. A standard deltopectoral approach was used for surgical exposure. The subscapularis tendon is first identified and sutured with a single No. 2 absorbable braided suture; the four edges of the tendon are marked (rotator interval, bicipital groove, circumflex vessels and conjoint tendon) and then it is released from its humeral insertion using an electrocautery device. At this point, the fracture pattern becomes clearly identifiable. When the lesser tuberosity is involved in the fracture, subscapularis release facilitates independent mobilization and reduction of the tuberosities, allowing reconstruction of the proximal humeral metaphysis before stem implantation. This reconstructed metaphyseal envelope provides more reliable anatomical references for stem positioning and humeral height, resembling the conditions of elective shoulder arthroplasty. Furthermore, subscapularis release facilitates subsequent glenoid exposure, minimizing excessive manipulation of the reduced fragments and the risk of loss of reduction or further fragmentation. The fragments are reduced and stabilized using a Bernhard towel clamp, after which two or more metallic K-wires are employed to secure the tuberosities (Figure 2).

**Figure 3:**
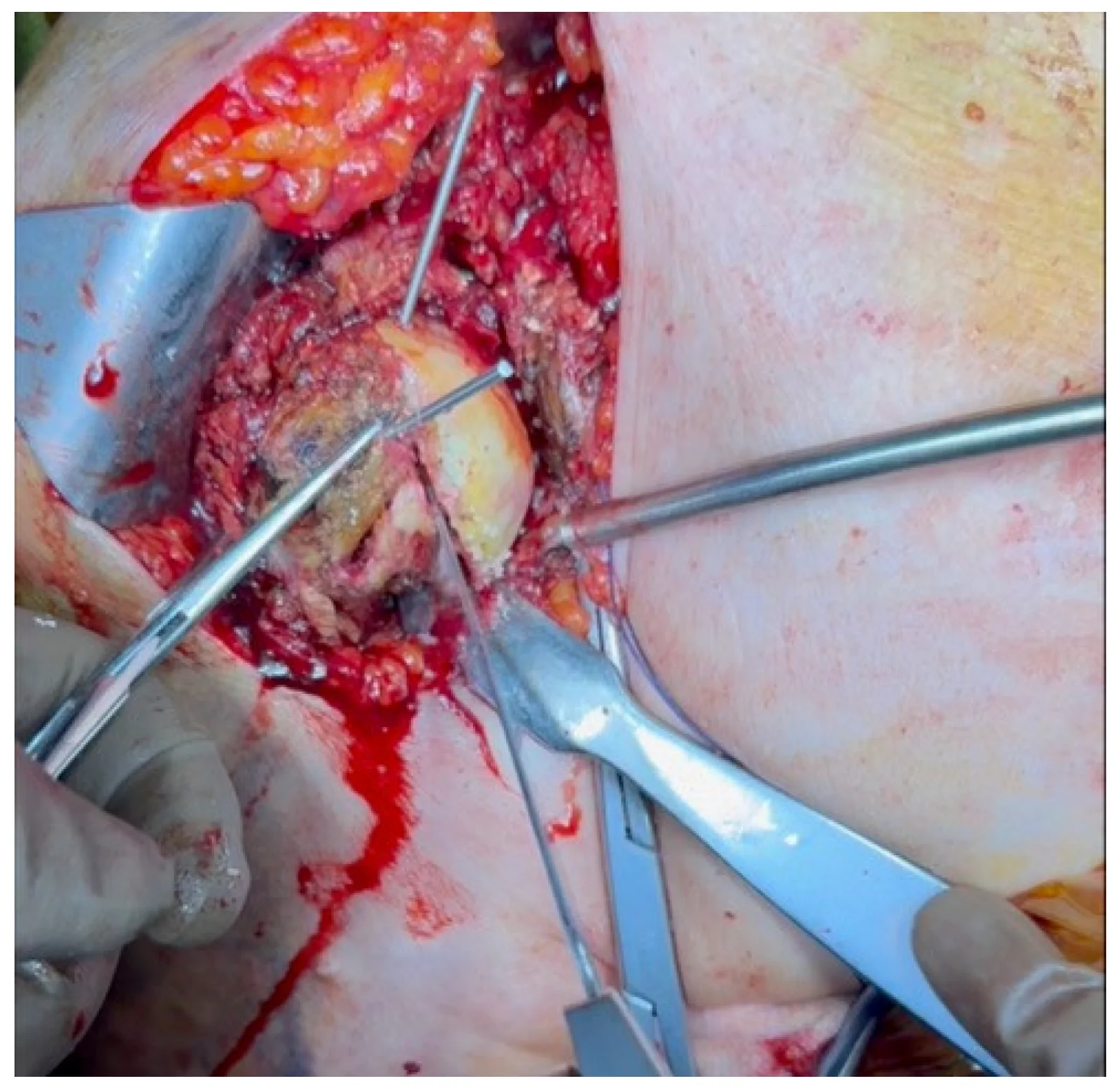
A precise resection of the humeral head is carried out using an oscillating saw.

This preliminary step is critical for restoring the native anatomy of the proximal humerus and preparing the tuberosities to “Hug” the stem, thereby initiating the replacement procedure. Once fixation is achieved, the humeral head is gently dislocated from the joint, without disrupting the fracture alignment. Using an oscillating saw, a precise humeral head resection is performed either with an extramedullary cutting guide or freehand technique; the latter is the author’s preferred method due to the presence of K-wires, which can interfere with the positioning of the guide (Figure 3).

**Figure 4:**
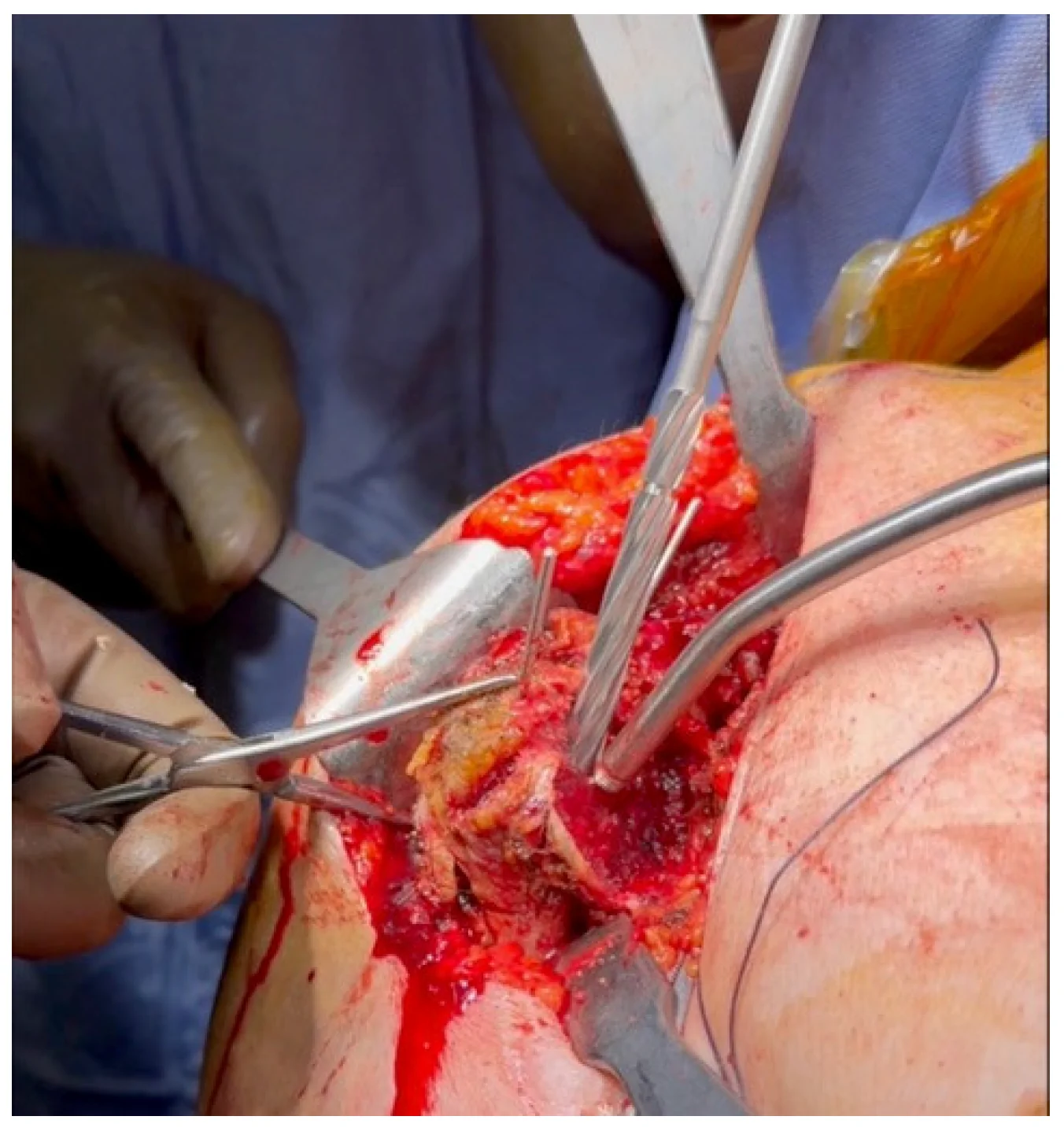
Initial identification of the diaphyseal canal is followed by progressive reaming.

**Figure 5:**
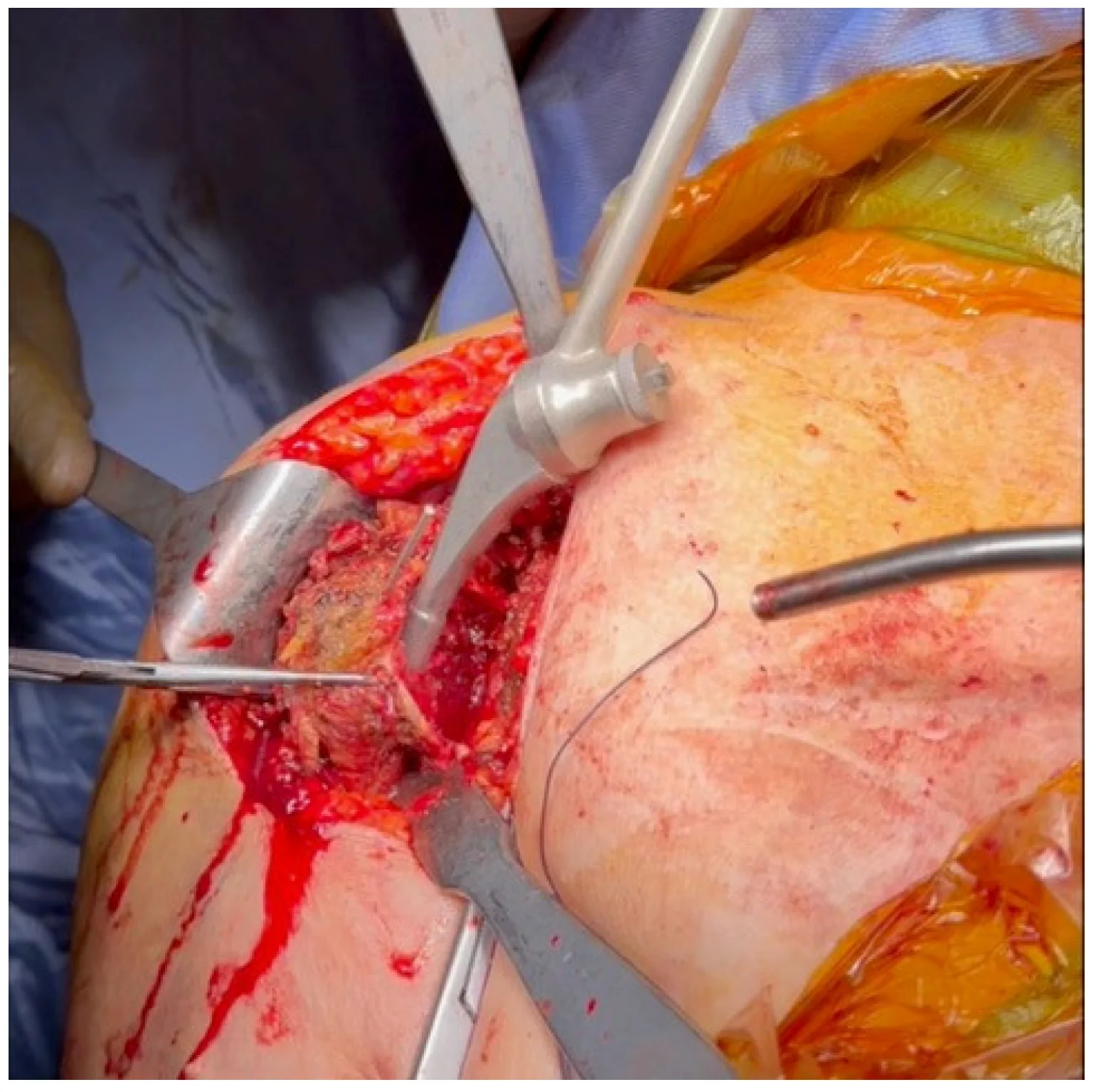
A trial broach with adequate and stable fixation is reached and left inside the humeral canal.

The diaphyseal canal is first identified and then progressively reamed (Figure 4) and broached until a trial broach with adequate and stable fixation is reached and left inside the humeral canal (Figure 5).

**Figure 6:**
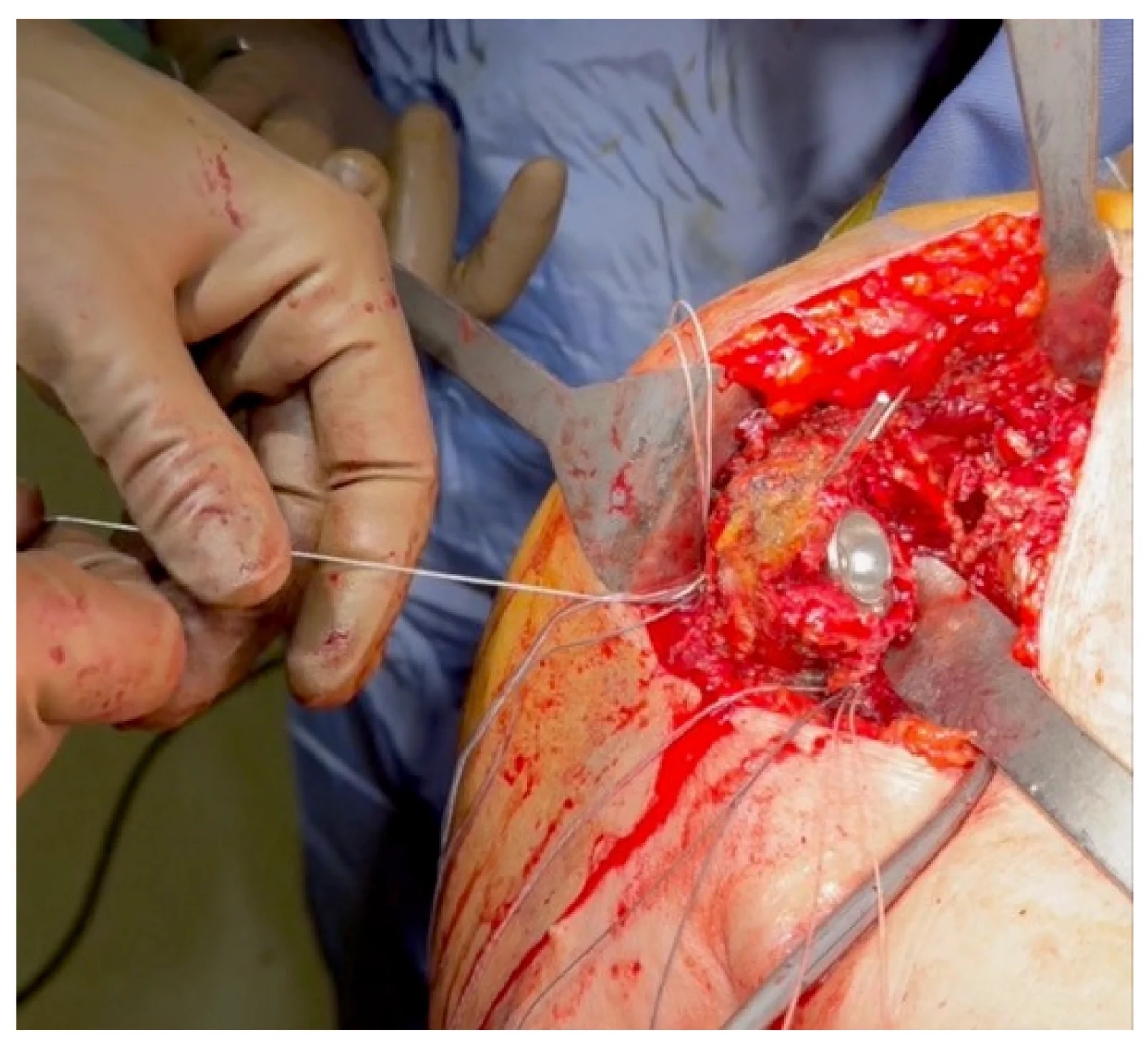
Three doubled suture-tape cerclages are passed through the shuttle wire, creating loops that emerge at the calcar, with both ends of each wire exiting through the tuberosities. The wires are positioned at varying levels along the tuberosities to optimize the distribution of the compressive forces generated by the “Hug” construct.

A Dechamps wire passer, preloaded with a 2-0 PDS suture used solely as a shuttle, is introduced anteriorly at the level of the calcar and advanced posteriorly to exit near the tuberosities. The Dechamps is removed and three duplicated Suture-tape Cerclage^®^ (Arthrex, Naples, FL, USA) are passed through the shuttle wire to form a loop emerging from the calcar and the two ends of each wire exiting from the tuberosities (Figure 6).

**Figure 7:**
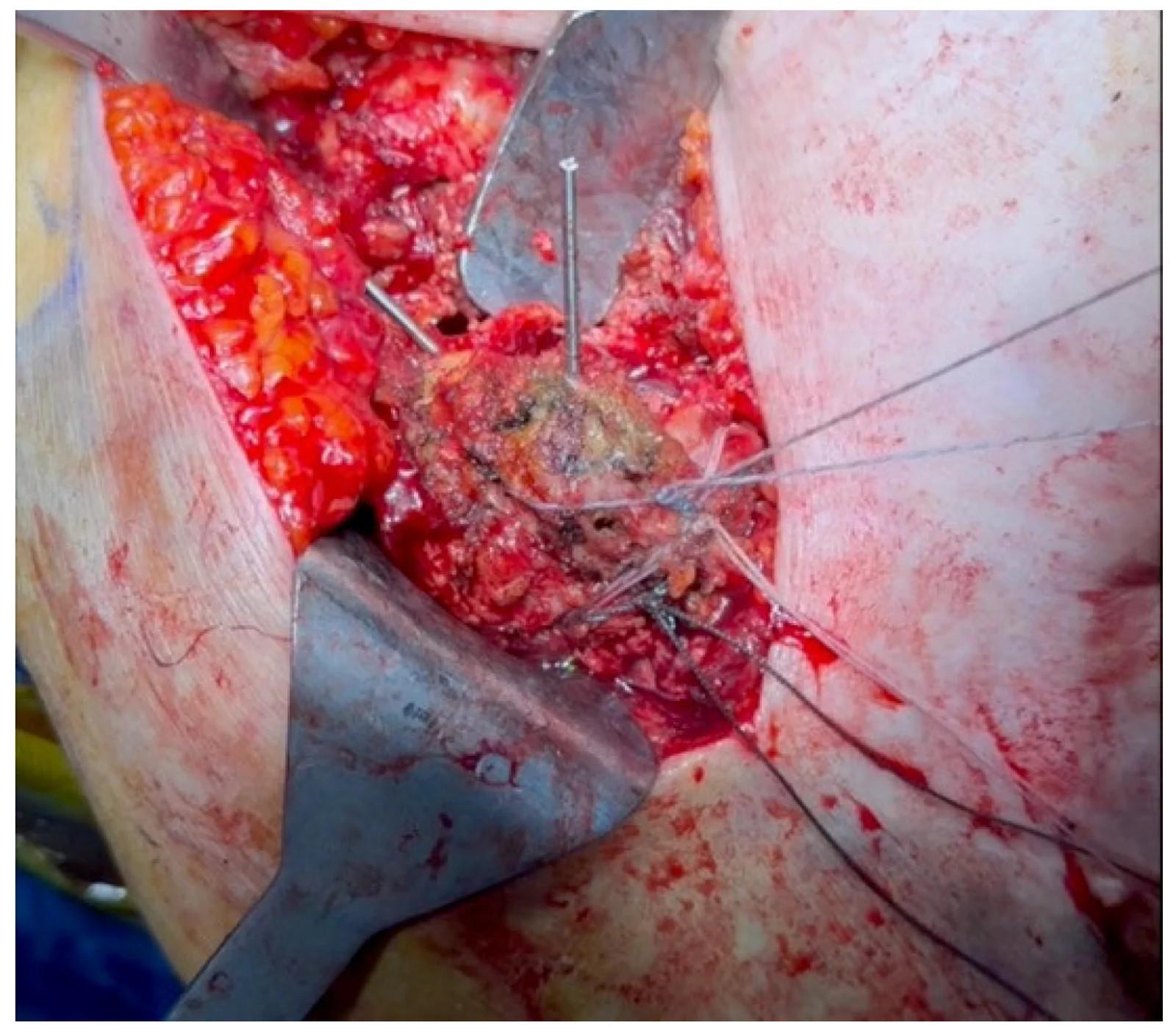
The doubled suture-tape cerclages are then secured using Nice knots to complete the “Hug” configuration.

**Figure 8:**
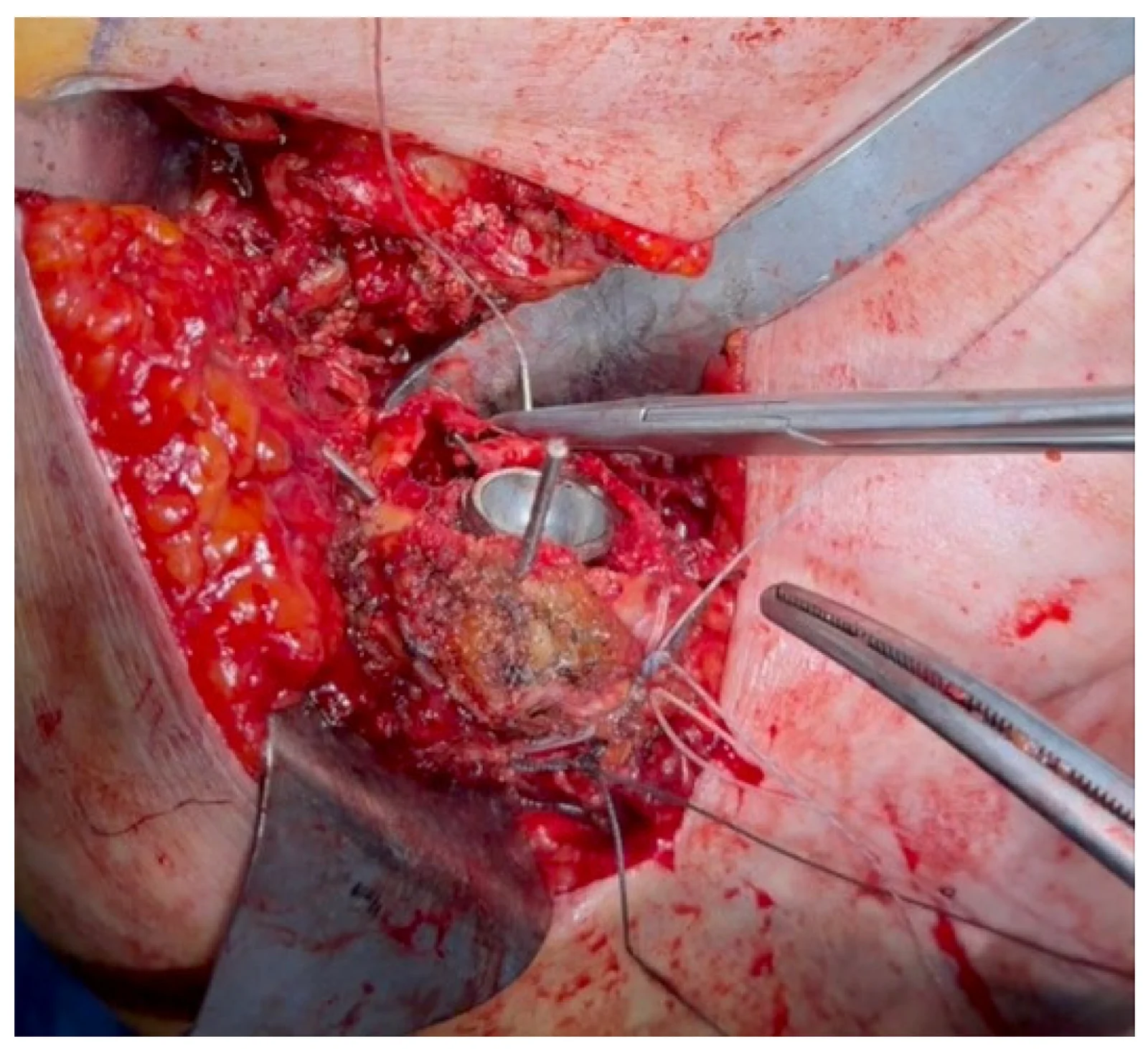
The suture ends, along with their attached needles, are intentionally left in place to facilitate subsequent reattachment of the subscapularis tendon.

The sutures are positioned at different levels along the tuberosities to optimize the distribution of compressive forces generated by the cerclage. They are subsequently secured to complete the “Hug” configuration using Nice knots (Figure 7), with the suture ends and attached needles intentionally left in place, as these will later facilitate reattachment of the subscapularis tendon (Figure 8).

**Figure 9:**
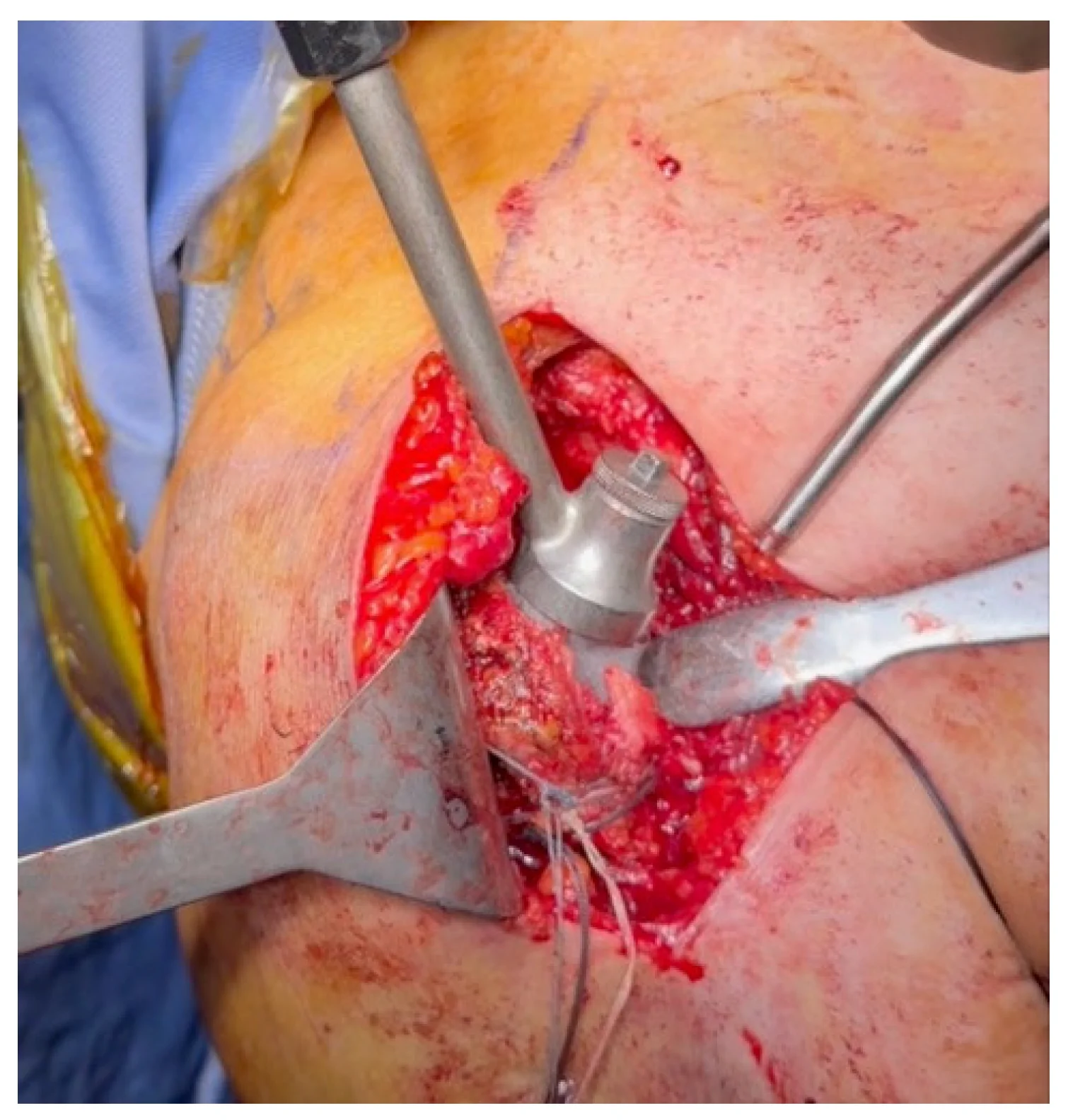
Following removal of the humeral trial components, the definitive cementless humeral stem is implanted.

**Figure 10:**
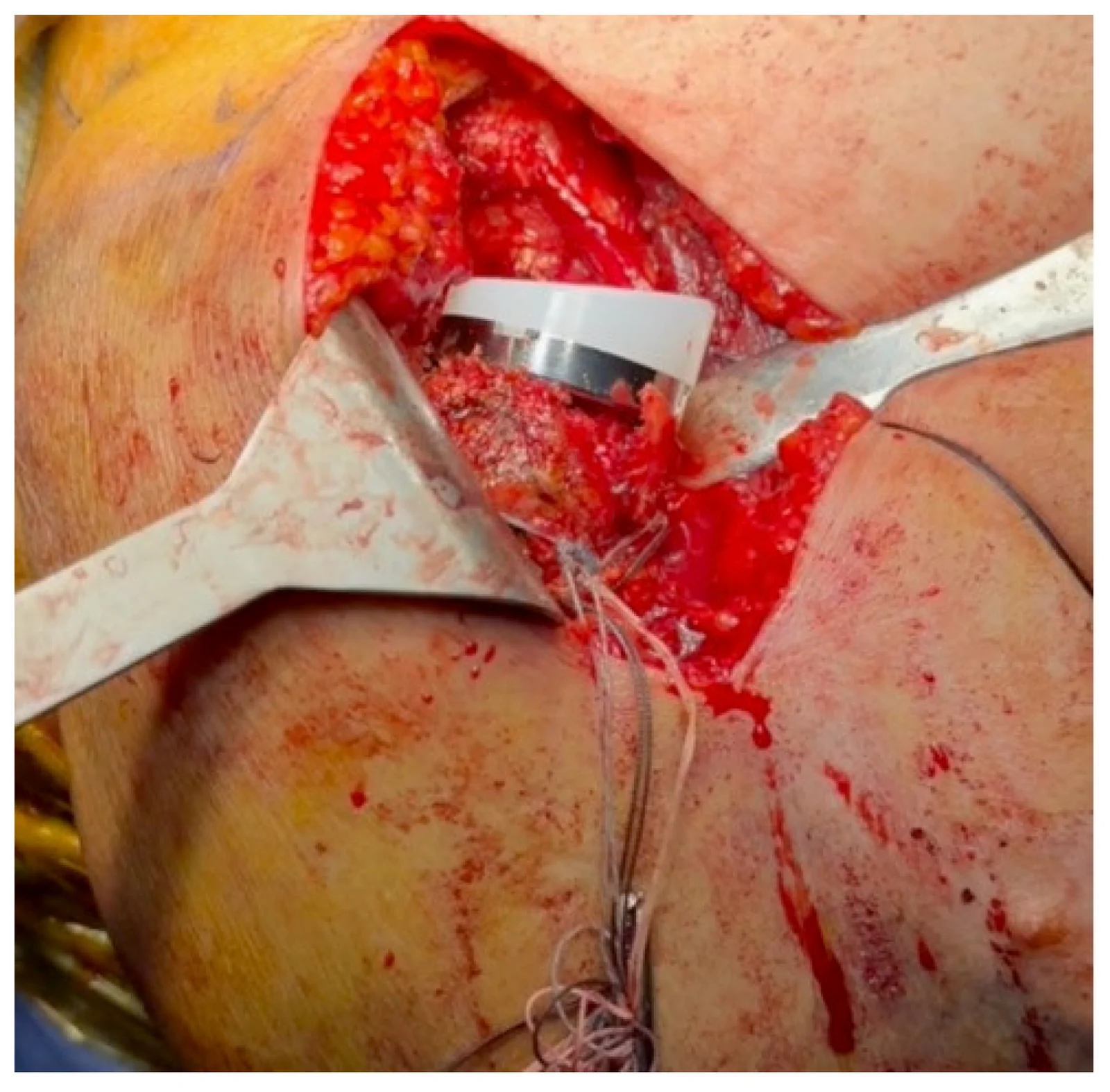
The humeral tray and polyethylene liner are inserted, followed by final impaction of the humeral prosthesis to achieve stable metaphyseal fixation.

All procedures were performed using the Equinoxe Reverse 145° shoulder system (Exactech, Inc., Gainesville, FL, USA). The humeral component consisted of a cementless, press-fit stem with an onlay configuration, allowing metaphyseal fixation while preserving the integrity of the metaphyseal cancellous bone. In each case, the smallest stem size providing adequate rotational stability was selected intraoperatively. When indicated, autologous cancellous bone grafting was employed before implantation of the definitive humeral stem to enhance metaphyseal fixation. With the humeral stem already in place, a trial humeral tray is inserted onto the stem to protect the proximal humerus during glenoid exposure and preparation. Glenoid component implantation is carried out using standardized surgical techniques, with glenoid preparation performed in accordance with the preoperative planning. Once the glenoid component has been implanted, attention is redirected to the humerus. The trial humeral metaphysis and liner are inserted to allow for temporary joint reduction. After confirming satisfactory stability through standard intraoperative tests, the components are dislocated again. The humeral trial components are removed, and the definitive humeral cementless stem is inserted, achieving stable metaphyseal fixation (Figure 9 and Figure 10).

**Figure 11:**
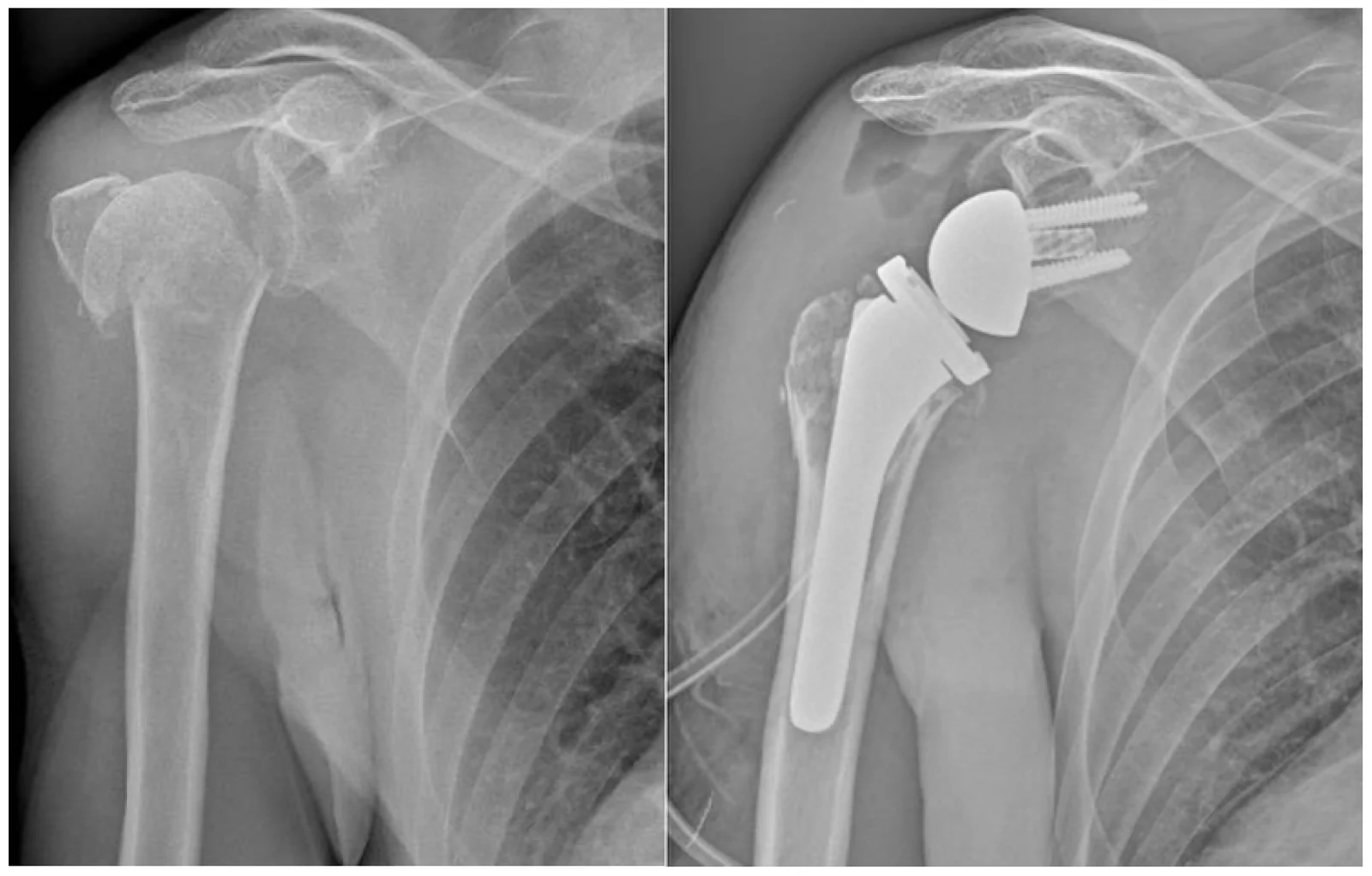
Preoperative and postoperative radiographs.

Following reduction of the definitive implant, the humeral stem functions as an intramedullary stabilizing device, ensuring proper fracture alignment and support across multiple anatomical planes. Despite being applied in a fracture scenario, the stem achieves reliable metaphyseal fixation comparable to that of a primary arthroplasty, without the need for cemented fixation. Figure 11 illustrates the preoperative and postoperative radiographs of the representative humeral fracture shown in the intraoperative figures.

The previously placed sutures used to secure the tuberosities are then employed to perform the reattachment of the subscapularis tendon, aiming to prevent loss of internal rotation range of motion (Table 1).

### 2.5. Rehabilitation Protocol

The operated arm is placed in an abduction and in neutral rotation brace for the first two weeks to reduce mechanical stress on the tuberosities, promote their anatomical healing, and protect the implant. With the exception of the type of brace employed during the first two postoperative weeks, the rehabilitation protocol following RSA using the Hug Technique is based on a standard fast rehabilitation protocol and is summarized in Table 2 [22].

## 3. Results

A total of 14 patients (14 shoulders) were included in the final analysis. The cohort consisted of 11 females and 3 males, with a mean age of 74.8 years (range, 67–86 years). The average follow-up duration was 14.2 months (range, 12–19 months). Six patients (42.8%) presented with three-part proximal humeral fractures (four involved the greater tuberosity and two involved the lesser tuberosity), while 8 patients (57.2%) sustained four-part fractures.

At final follow-up, patients demonstrated satisfactory functional recovery. The mean Constant score was 68.4 ± 9.2 and the mean ASES score was 81.6 ± 10.5. Mean active forward flexion reached 132° ± 18°, mean abduction was 118° ± 15°, and mean external rotation at 0° abduction was 28° ± 9°. Internal rotation corresponded to a mean vertebral level of L3.

Radiographic evaluation at final follow-up demonstrated anatomical or consolidated tuberosity healing in 11 patients (78.5%). Specifically, anatomical healing was observed in 9 patients, malunion in 2 patients, and tuberosity resorption or nonunion in 3 patients. No cases of humeral stem loosening or subsidence were identified.

Postoperative complications occurred in 2 patients (14.2%). One patient developed an early deep periprosthetic infection at 2 weeks postoperatively, which was successfully treated with Debridement, Antibiotics and Implant Retention (DAIR). Another patient, among the three who developed tuberosity resorption, experienced postoperative anterior dislocation requiring revision surgery, with upsizing of both the humeral tray and the glenosphere. No neurological complications or periprosthetic fractures were recorded during the follow-up period.

At final follow-up, 13 of 14 implants remained free from revision, corresponding to an implant survivorship free from revision of 92.9%, while one patient (7.1%) required revision surgery because of postoperative anterior dislocation.

The main characteristics of the patients included in the study are summarized in Table 3.

## 4. Discussion

The main finding of the present study is that the Hug Technique represents a feasible approach for cementless reverse shoulder arthroplasty in acute three- and four-part proximal humeral fractures, with promising preliminary functional outcomes and a high rate of tuberosity healing. In this series, anatomical healing or malunion of the tuberosities was observed in 11 of 14 patients (78.5%), while no cases of humeral stem loosening or subsidence were recorded. Nevertheless, two major complications occurred, including one deep periprosthetic joint infection and one anterior dislocation requiring revision surgery. These findings should therefore be interpreted cautiously given the small sample size, absence of a control group, and single-surgeon design.

The established role of reverse shoulder arthroplasty in the management of complex three- and four-part proximal humeral fractures in elderly patients has been supported by previous clinical studies. Iacobellis et al. [23] reported favorable clinical outcomes following RSA in elderly patients with complex proximal humeral fractures, further supporting its effectiveness as a reliable treatment option in this challenging population. Nevertheless, functional recovery, tuberosity healing, implant stability, and postoperative complications remain important considerations when evaluating different surgical approaches. In the present series, the Hug Technique achieved a mean Constant score of 68.4, a mean ASES score of 81.6, and a mean active forward flexion of 132°, with anatomical or consolidated tuberosity healing observed in 78.5% of patients. No humeral stem loosening or subsidence was detected at a mean follow-up of 14.2 months. Unlike conventional fracture-specific reconstruction techniques, which frequently require tuberosity mobilization and extensive manipulation of the proximal humeral fragments, the Hug Technique allows preliminary anatomical reduction and stabilization of the tuberosities before humeral head resection, thereby preserving metaphyseal bone stock and maintaining the surrounding soft-tissue attachments. Furthermore, the use of circumferential suture-tape cerclage provides compression of the tuberosities around a standard cementless press-fit stem, potentially promoting biological healing while maintaining primary implant stability. This approach may represent a valuable alternative to conventional cemented RSA and other cementless reconstruction techniques, particularly when sufficient metaphyseal bone stock is available. However, the occurrence of two major postoperative complications in our cohort, including one deep periprosthetic infection and one anterior dislocation requiring revision surgery, highlights the importance of careful patient selection and surgical execution. Although the preliminary results are encouraging, the absence of a comparative control group and the limited follow-up prevent definitive conclusions regarding the superiority of the Hug Technique over established RSA techniques.

The literature presents conflicting evidence regarding clinical outcomes, active range of motion (ROM), radiographic results, and complication rates between patients undergoing RSA for elective indications (RSA-E) and those treated for RSA for fracture (RSA-F). Paras et al. reported that RSA-F performed for acute three- and four-part proximal humeral fractures results in overall poorer clinical outcomes and reduced active ROM compared to RSA performed for elective indications such as cuff tear arthropathy, massive irreparable rotator cuff tears, and osteoarthritis with deformity [24].

Conversely, data from the New Zealand Joint Registry at 10-year follow-up indicate comparable long-term survivorship and functional outcomes between RSA-F and RSA-E [1]. Similarly, Jenkins et al., in a study involving 384 patients who all received the same prosthetic design, found no significant differences in clinical or radiographic outcomes at a minimum of 5 years’ follow-up between patients undergoing RSA-F and those treated for degenerative conditions [25].

The “Hug Technique” offers the advantage of employing a stem with metaphyseal porosity, as it is designed for RSA-E, and this allows for enhanced biological fixation without the need for cementation. Furthermore, by avoiding osteotomy to mobilize the tuberosities, the structural integrity of the periosteum and its associated soft tissues is maintained, which promotes optimal biological healing and tissue regeneration.

Great efforts have been made to understand how prosthetic design influences tuberosity healing; the potential advantages of using fracture-specific stems in RSA-F have been widely explored. These implants incorporate several design modifications intended to enhance tuberosity healing, including lateral fins with suture holes for robust tuberosity fixation, a proximal metaphyseal window to facilitate bone growth across the fracture site, and a coated metaphyseal region to promote osseous integration between the tuberosities and the humeral shaft [26].

The clinical superiority of fracture-specific stems compared to standard humeral stems remains inconclusive, as retrospective studies and systematic reviews have yielded conflicting evidence regarding their impact on tuberosity healing and overall shoulder function [27,28,29]. Nevertheless, Imiolczyk et al. demonstrated that conventional humeral stems can achieve comparable clinical outcomes to modern fracture-specific designs. Both cohorts exhibited similar qualitative and quantitative tuberosity union rates, suggesting that the additional design features of fracture stems may not provide a clear advantage in promoting healing. Furthermore, the only functional parameter with a notable difference was active forward flexion, which was superior in the group treated with conventional stems [30]. The reason for this isolated difference remains unclear. The authors suggested that it may have been influenced by differences in follow-up duration rather than by an intrinsic biomechanical advantage of the conventional stem.

In the present series, tuberosity healing also appeared to be associated with functional recovery. Patients with tuberosity resorption/nonunion showed poorer functional outcomes, with lower Constant and ASES scores at final follow-up compared with patients with preserved tuberosity healing. However, given the small number of patients in this subgroup, these findings should be interpreted descriptively and no definitive conclusions regarding this association can be drawn.

Humeral inclinations have also been studied by O’Sullivan et al., who demonstrated that RSA-F with a 135° prosthetic inclination is associated with significantly higher rates of tuberosity healing, 83% compared to 69% with a 145° inclination and 66% with a 155° inclination [31].

In the present series, all patients were treated using the same standard cementless press-fit humeral stem, rather than a fracture-specific stem. This implant choice is consistent with the rationale of the Hug Technique, which aims to preserve metaphyseal bone stock while achieving stable biological fixation. In this context, previous studies have investigated the potential advantages of cementless humeral fixation in RSA for proximal humeral fractures [32].

Concurrent with advances in prosthetic design, significant efforts have been directed towards enhancing the outcomes of RSA-F through the implementation of novel surgical techniques and meticulous operative strategies specifically aimed at facilitating tuberosity healing and optimizing prosthetic stability.

Russo et al. presented a comparative analysis of clinical outcomes between two surgical techniques for tuberosity reconstruction during RSA-F in elderly patients [33]. The first technique, known as the “Puzzle Pieces Reconstruction” (PPR), involves identifying and anatomically reassembling the fracture fragments to restore the correct tuberosity height in relation to the prosthetic humeral head, followed by fixation with non-absorbable osteo-sutures, based on principles inspired by the Boileau method [7]. The second, referred to as the “Bone Collar and Tie” (bCAT) procedure, entails creating a collar graft by shaping the resected humeral head into a crescent form and positioning it between the prosthetic humeral body and the greater tuberosity to fill the lateral-posterior void around the implant. In a comparative study of two cohorts of 20 patients each, the authors reported superior functional outcomes in the bCAT group, particularly in terms of external rotation. This improvement was attributed to more effective healing and optimal anatomical positioning of the tuberosities in elderly patients, which allowed better preservation of residual rotator cuff function without requiring modifications to the prosthetic design. Notably, both techniques differ from the approach described in the present study, as they require resection of the humeral head at the beginning of the procedure and the performance of osteotomies to mobilize the tuberosities and assess proximal humeral bone loss.

Formaini et al. introduced the “black and tan” technique, a hybrid cementation and impaction grafting method that employs autologous cancellous bone graft to create a biologic interface between the proximal cement mantle and the tuberosity repair region in RSA-F, thereby avoiding proximal thermal bone necrosis [34].

A recent systematic review and meta-analysis by Kaiser et al. [35], including 1797 RSAs for proximal humeral fractures, reported a significantly higher greater tuberosity healing rate with uncemented press-fit stems (80%) compared with the black-and-tan technique (70%) and fully cemented stems (61%). These findings support the potential biological advantages of cementless humeral fixation in promoting tuberosity healing.

Unlike techniques found in the literature, one of the main advantages of the “Hug Technique” is its ability to restore the correct height of the humeral stem, which helps to prevent both instability and an overly constrained implant and preserves deltoid tension, which is crucial for improving ROM [36]. This is achieved through a humeral head osteotomy performed after obtaining adequate reduction of the fracture fragments, in a manner similar to that used in RSA-E. This technique preserves the anatomical landmarks of the pre-injury shoulder and maintains appropriate soft tissue tension. This approach eliminates the need for intraoperative fluoroscopy, and the surgeon does not need to rely on predefined measurements, such as those described by Ponce et al., who recommend positioning the lateral border of the humeral stem 5.6 cm proximal to the pectoralis major tendon [37]. Performing the osteotomy only after adequate reduction ensures accurate humeral distalization and contributes to improved prosthetic stability.

The acute timing of RSA may also have contributed to the favorable functional outcomes observed in the present study. Caldaria et al. [4] reported better functional outcomes in patients undergoing primary RSA for proximal humeral fractures compared with salvage RSA after failed previous treatment. Therefore, the acute management of all fractures in the present cohort should be considered a potential contributing factor to the observed outcomes. However, acute treatment is an inherent component of the indication for the Hug Technique, as the procedure relies on preservation and reduction of the native fracture fragments, tuberosities, and surrounding soft-tissue relationships; consequently, its application is not intended for delayed fracture sequelae.

Despite these potential advantages, the Hug Technique may also present some limitations and theoretical risks. Preservation and reduction of the proximal humeral fragments may be technically demanding in highly comminuted fracture patterns, while achieving adequate primary stability with a cementless stem may be challenging in patients with poor bone quality. In such cases, inadequate fixation could theoretically increase the risk of stem subsidence or intraoperative fracture. Furthermore, the technique requires careful preservation of the tuberosities and surrounding soft tissues throughout the procedure, which may increase its technical complexity. Finally, as the technique was performed by a single experienced shoulder surgeon, its learning curve and reproducibility among different surgeons remain to be established.

This study presents several limitations that should be considered when interpreting the results. The retrospective nature of the analysis introduces a potential risk of selection bias and limits the ability to establish causal relationships. Although all procedures were performed by the same experienced surgeon, ensuring consistency in surgical technique and follow-up, this may reduce the generalizability of the findings to other clinical settings or less experienced surgeons.

The relatively small sample size may have limited the statistical power to detect subtle differences in clinical outcomes or complication rates. Furthermore, the absence of a control group treated with alternative reconstructive strategies or cemented stems limits the possibility of direct comparisons and prevents determination of whether the “Hug Technique” is superior to other surgical methods.

The clinical implications of the present study lie in the potential application of the Hug Technique as a standardized surgical strategy for managing complex proximal humeral fractures requiring reverse shoulder arthroplasty. By restoring proximal humeral anatomy before prosthetic implantation, this approach may help surgeons achieve more consistent component positioning and tuberosity reconstruction, potentially reducing technical variability in fracture arthroplasty. This may be particularly relevant in challenging fracture patterns, where anatomical landmarks are frequently disrupted and accurate humeral stem positioning can be difficult. Furthermore, the proposed surgical sequence may facilitate intraoperative decision-making and provide a more reproducible approach to fracture management. Although the preliminary clinical outcomes are encouraging, further comparative studies are required to determine whether these technical advantages translate into improved functional outcomes and reduced complication rates.

## 5. Conclusions

The “Hug Technique” represents a feasible surgical strategy for cementless reverse shoulder arthroplasty in acute three- and four-part proximal humeral fractures. By preserving metaphyseal bone stock and avoiding tuberosity osteotomy, this technique aims to optimize biological healing conditions while allowing stable metaphyseal fixation with a standard press-fit stem. The preliminary clinical and radiographic findings observed in this small case series are promising, particularly with regard to functional recovery and tuberosity healing. However, given the limited sample size, the absence of a control group, and the occurrence of major complications, these findings should be interpreted with caution. Further prospective comparative studies involving larger cohorts and longer follow-up are required to assess the reproducibility, safety, and potential clinical benefits of this technique.

## Figures and Tables

**Table 1:** Key pearls and pitfalls of the Hug Technique for reverse shoulder arthroplasty in complex proximal humeral fractures.

Pearls	Pitfalls
Use the bicipital groove as a key landmark for tuberosity orientation	Misinterpreting the fracture pattern → inaccurate reduction
Stabilize the fragments early with clamp + K-wires before head resection	Dislocating the humeral head before stabilization → loss of anatomical references
Keep the trial stem in place to preserve alignment during canal preparation	Over-reaming → loss of press-fit and poor stem stability
Place cerclage sutures at multiple vertical levels to distribute forces	Malpositioned or overtightened cerclages → tuberosity necrosis or loosening
Reduce the fracture before head osteotomy to restore humeral height accurately	Inadequate restoration of humeral height → poor deltoid tension and joint instability

**Table 2:** Postoperative rehabilitation protocol following reverse shoulder arthroplasty using the Hug Technique.

Postoperative Phase	Immobilization/Protection	Rehabilitation
Postoperative day 1–14	Abduction brace with the arm in neutral rotation	Active-assisted mobilization of the elbow, wrist, and hand; cervical spine mobilization. Passive shoulder mobilization in the supine position: forward flexion ≤ 90°, abduction ≤ 45°, and external rotation ≤ 20° in the scapular plane.
Day 15–44	Brace discontinued	Progressive increase in passive ROM. Introduction of active-assisted shoulder mobilization, pendulum exercises, scapulothoracic mobilization, and scar massage.
From day 45	—	Active shoulder mobilization. Gradual progression to isotonic strengthening and proprioceptive retraining.
From 3 months	—	Strengthening against resistance, once full passive ROM has been restored and joint stability is clinically evident.

**Table 3:** Demographic, clinical, surgical, and radiographic characteristics of the patients included in the study.

Patient	Surgery	Age	Sex	Neer	Injury–Surgery (d)	Graft	FU (mo)	Tuberosity Status	Constant	ASES	Complication
1	24-June	67	F	4-part	4	Yes	19	Anatomical	80	96	None
2	24-June	72	F	3-part	3	No	17	Anatomical	77	91	None
3	24-June	69	M	4-part	5	Yes	16	Anatomical	74	88	None
4	24-Aug	74	F	4-part	4	Yes	15	Malunion	65	75	None
5	24-Aug	78	F	3-part	6	No	15	Anatomical	72	85	None
6	24-Sep	71	F	4-part	5	Yes	14	Anatomical	76	90	None
7	24-Sep	75	M	3-part	3	No	14	Anatomical	70	84	None
8	24-Oct	80	F	4-part	7	Yes	14	Resorption/nonunion	55	65	Anterior dislocation; revision
9	24-Oct	73	F	3-part	4	No	13	Anatomical	71	86	None
10	24-Nov	77	F	4-part	6	Yes	13	Malunion	63	75	None
11	24-Nov	68	F	3-part	3	No	13	Anatomical	79	92	None
12	24-Dec	86	F	4-part	8	Yes	12	Resorption/nonunion	53	63	PJI; DAIR
13	24-Dec	76	M	3-part	5	No	12	Anatomical	69	83	None
14	25-Jan	81	F	4-part	4	Yes	12	Resorption/nonunion	54	69	None

## Data Availability

The dataset is available on request from the authors.
